# Cerebro Spinal Anæmia and Convulsions

**Published:** 1875-03

**Authors:** W. W. Munson


BUFFALO

Medical and Surgical Journal.

VOL XIV. MARCH, 1875. No. 8.

### Original Communications.

:o:

ART. 1.—*Cerebro-Spinal Anaemia and Convulsions.* By W. W. MUNSON, M. D. Read before the Central New York Medical Association, December 15th, 1874.

Miss D.——, aged 22, school teacher. Found her in bed, with headache, vertigo, nausea and vomiting; all these symptoms notably increased on rising from the recumbent position. Face pale, lips colorless, conjunctivæ excessively white, pupils widely dilated, contracting but sluggishly on exposure to strong light, retinæ extremely sensitive. No pain or tenderness in any part of body, save the cephalagia.

Diagnosis: Cerebral Anæmia, produced by the exhaustion of excessive mental exertion. Treatment: Rest, concentrated and easily digested nourishment every four hours when awake, bismuth and oxalate of cerium before eating until nausea and vomiting are controlled, pyrophosphate of iron and sulphate of strychnia in infusion of gentian and whiskey after eating. Extra amount of whiskey at night to relieve the insomnia. Nourishment and medicine well retained from the first. After two days, food and tonic given in the usual manner, three times a day. Improvement rapid; cure complete in one month.

Mrs. D., aged 41, mother of two children; general appearance that of a stout, healthy woman of 150 pounds weight. Her chief complaint is of a severe pain in right hand and arm. This pain is not constant, but occurs in paroxysms which are specially violent on going to sleep, the pain waking her as soon as she commences to drowse. Fingers are numb; reports partial paralysis of right lower extremity, accompanied with pain, at each menstrual period, states that the foot drags and trips, approaching the appearance of one suffering from hemiplegia. This condition has lasted several months, but she has not been under treatment; only applies now for relief from pain. Face and lips are pale, pupils are dilated, responding to light sluggishly. She has never suspected any spinal difficulty, has felt no pain or soreness in back, but on examining spine I find she flinches and complains of pain, on pressing spinous processes of seventh cervical and first four dorsal vertebræ. Tenderness not elecited in any other part of spine. Pain was relieved and sleep obtained by the use of opiates, but the usual unpleasant effects of this drug appearing next day, no more of it was used.

Examination per vagina reveals chronic vaginitis. Diagnosis: Cerebro-spinal anæmia, the spinal element predominating. She is put upon the same constitutional treatment as the preceding case, with solution of sulphate of zinc and carbolic acid for vagina. If she is sleepless, give her whiskey, if in pain give her whiskey, if dizzy give her whiskey; in fact all the alcohol is given her that can be borne. Recovery in this case was rapid.

From several similar cases that have come under my observation, these two are selected as the text, and cerebro-spinal anæmia as the theme of this paper.

Although the condition so long unfortunately known as "spinal irritation" is not recognized as a distinct disease by many of our best authorities, I cannot but believe that we do sometimes meet a case presenting nervous symptoms not described by medical writers except those who treat of "spinal irritation" as an affection separate and distint from general anæmia, hysteria or exhaustion. To be sure the symptoms are very variable and inconstant, even more so than those of that most capricious of nervous maladies which we still ignorantly term hysteria.

The prominent, essential, even pathognomonic symptom of spinal anæmia is tenderness over one or more points of the spinal column. This tenderness is always increased by pressure, and may or may not be previously complained of by patient. In four of my cases it was not even thought of by the patient till I discovered it by pressing on spinous process. In one of these cases pressure not only produced pain at point of tenderness, but also pain and numbness in right arm and hand. In another the usual left infra-mammary stitch and a sickening faint feeling were produced, so great that she come near falling to the floor. In another the pain in the back, arms and chest were so great that she was several hours recovering from the effects of only slight pressure. Of so much importance is this symptom that whenever a train of symptoms present themselves, clearly belonging to the nervous system, but not answering to the description of any well known disease, the spine should always be examined. To be sure, spinal tenderness is often (some insist always) found in hysteria. But in uncomplicated spinal anæmia we never find one of those incongruous combinations of symptoms known as the hysteric fit. Besides, in hysteria, there is almost invariable the hypograstric tenderness, pressure over left ovary will often temporarily relieve all symptoms at once. I have taught one of my hysterical patients to relieve her "globus hystericus," giggling and crying, by hard pressure with the fist down on left ovary. She has no spinal tenderness

Around this symptom, spinal tenderness, and apparently depending upon the pathological condition which produces it, cluster all the other symptoms of spinal anæmia, such as pain, nausea and vomiting, with other dyspetic symptoms, cough, dyspnœa, palpitation of the heart, irritability of the bladder, syncope, hiccup, dysphagia, anæsthesia, hyperæsthesia, paralysis, convulsions, spasms, fibrillary twitchings, contraction, numbness, vertigo, noises in the ears, disturbance of vision, aphonia, epilepsy, and chorea—the different symptoms and combination of symptoms depending greatly upon the seat of spinal tenderness. Thus, when the irritation exists in the cervical region only, with which condition, I believe cerebral anæmia is usually associated; there will be vertigo, headache, noises in the ears, disturbances of vision, sense of fullness and constriction across forehead, derangement of sleep, usually insomnia, more rarely somnolence with unpleasant dreams and nightmare, neuralgic pains in face, neck, shoulders and arms, fibrillary twitchings and spasms of muscles of face and neck, contractions of some of the muscles supplied by cervical nerves, especially the flexors of hands and arms. Paralysis may also occur in these muscles, aphonia and hiccup, nausea and vomiting. General chorea has also been noticed; while the mind is almost sure to be more or less affected. It is not to be understood that all of these symptoms will ever exist in any one case, but all of these symptoms have been noticed in different cases where the cervical region was only affected.

When the dorsal region only is affected the most prominent symptoms are connected with the viscera. The stomach is always more or less deranged, gastralgia, nausea and vomiting, pyrosis and flatulence being very constant symptoms. Syncope, depression, cough, hiccup, dyspnœa and intercostal neuralgia, especially the left infra-mammary stitch, may all be looked for. No spasms, contractions or paralysis have as yet been noticed, as far as I am informed, in anæmia of dorsal region only. When the lumbar region alone exhibits spinal tenderness, the symptoms are all connected with the organs supplied by nerves given off from the lumbar spine. There may be pain contractions, spasms or paralysis in lower extremities, pain in bladder, rectum, ovaries and uterus, and incontenence of urine.

We may not hope, however, to often see a case in which there will be tenderness over any one region alone, with the corresponding symptoms.

It is when different regions of the spine are affected at the same time, as is usually the case, the symptoms become complicated. But there need be no confusion, for the connection of different symptoms and groups of symptoms with their tenderness in different regions of the spine, may always be observed if the anatomical distribution of the nerves be borne in mind, "one group of symptoms changing into another as the spinal tenderness becomes more marked in one region than another," (Radcliff.)

But it is where the whole spine is tender that the disease becomes more serious and obstinate. In such cases the hyperæsthesia is very extensive and acute, it being scarcely possible to press upon the most limited point without producing pain. In these comparatively rare cases we may expect paralysis, epilepsy, clonic and tonic spasms, prolonged muscular contractions, severe neuralgic pains in any part of the body, with any of the other symptoms enumerated as belonging to any particular local tenderness. I have seen but one such case. In this case the slightest pressure in any part of the spine would cause the patient to wince and cry out with pain radiating to parts supplied with nerves arising from the region to which pressure was applied. This was a case of great interest to me, as I long looked upon it as one of hysteria only; but when I considered the superior intelligence and calm judgment of the patient, the almost complete paralysis of right extremities, the severe, painful spasms, both tonic and clonic, the agonizing visceral neuralgias, and the long continued contraction of right anterior and posterior tibials, producing a firm and constant talipes varus which continues to a slight degree to this day—two years after her otherwise complete recovery; after these and many other considerations, I was led to believe that I had something more than hysteria to deal with.

As to the pathology of this disease, whether the sympathetic system of nerves be at fault or not, the best reason, among many others, for believing that it is dependant upon anæmia of the brain and spinal cord is this: "Those agents which are known to diminish the amount of blood in the spinal vessels invariably increase the severity of the symptoms due to spinal irritation, while they are as effectually lessened in intensity by remedies which tend to produce spinal hyperæmia." (Hammond).

Dr. C. B. Radcliff inclines to the belief that spinal irritation involves "capillary contraction and bloodlessness," "deficiency of blood and organic changes brought on by the part being starved for want of blood."

Dr. John P. Gray, of the Utica Asylum, in his paper read before the State Society, 1871, insists upon cerebral anæmia as a common cause of insanity.

With the view of the pathology of this affection, that it is dependent upon anæmia of the brain or cord, or both, the treatment, in any of its forms, suggests itself, namely: those agents that will supply these organs with a proper amount of healthy blood, and improve their nutrition. These we have in alcohol, strychnine, phosphorus, iron and electricity, good food, air, light and exercise with proper influences and associations being of course understood.

In obedience to the recommendations of all authorities that I have consulted on this subject, I blistered my first cases; but about two years ago, a lady come under my care with spinal irritation, who was not ill enough to suspend her household duties, and thinking a sore between the shoulders would be rather uncomfortable to labor under, I depended upon alcohol, iron and strychnine, recommending good nourishing food, and cautioning against overwork. She did well. Since then I have blistered but one case, and henceforth I shall not resort to the counter irritant till other means fail. If anæmia be the pathological condition in this affection, where is the indication for *counter* irritation? On the contrary, are not those agents indicated that *directly* stimulate the parts at fault? In those cases where benefit has been derived from irritation applied to spine, I suggest that the irritation, being applied so near the seat of disease, instead of acting as a *counter* irritant, *directly* increased the blood supply and nervous energy to the anæmic cord beneath. I have used electricity in only one case of this affection. In this case it failed to be of the least service, although it was long and faithfully continued. Nevertheless I have no doubt of its efficacy in this disease as well as in all others where nerve stimulation and nutrition are desired. In these days of electro-therapeutics, when every doctor, quack and regular, has his batteries, and when specialists in this department are curing every thing with the different "currents," from herpes and eczema to elephantiasis and paralysis; it may be presumptious to speak a depreciative word upon the subject. Still I cannot consider the warning which Dr. J. Russell Reynolds gives in his little work on the clinical uses of electrity, at all ill timed, when he cautions both students and practitioners to not overestimate the power of electricity in the diagnosis and treatment of disease. With

"country doctors" the *simplest* way is usually the *only* way available, whether it be the *best* way or not.

The prescription which I usually commence with in these cases, and which I feel safe to recommend, is a solution of pyrophosphate of iron and sulphate of strychnine in a strong infusion of gentian sweetening, and adding one fourth good whiskey, the proper dose being contained in a tablespoonful, to be taken three times a day after eating. This is easily made, pleasant to take, contains one of our best preparations of iron, and we can be positively sure of the amount of strychnine given, which is not always the case when we trust to any of the different fancy preparations of iron and strychnine or to either of the extracts of nux vomica. If the above fails we have the phosphate of zinc, pure phosphorus in many pleasant forms, the dilute phosphoric acid, the fluid and solid axtracts of nux vomica, a multitude of good preparations of iron, a long list of "wines and liquors," quinine, electricity and blisters, to fall back upon.

Of course if the exciting cause of the condition can be ascertained, it should, if possible, be removed. But this is not always easily discerned. Says Hammond, in summing up this matter; "Any cause capable of reducing the powers of the system may produce spinal irritation."

Morphine administered hypodermically is almost sure to relax the spasms or convulsions in a few minutes.

Convulsions in spinal irritation as elsewhere, being a symptom, not a disease, should always be treated with the same general principal in view: (1) Remove the cause if it can be discovered: (2) Apply those measures that will the most rapidly end safely, determine the blood to the brain and spinal cord.

To be sure we have epileptic convulsions, apoplectic convulsions, hysterical convulsions, puerperal convulsions, syphilitic convulsions, uræmic convulsions, centric convulsions, excentric convulsions, eclamptic convulsions, general convulsions, unilateral convulsions, local convulsions, convulsions from injuries about head or any other part of the body, with or without hæmorrhage, convulsions from tumors or abscess in or on brain or cord, worm fits, teeth fits, green apple fits—all sorts of convulsions, spasms and fits—but the weight of opinion among the most distinguished clinical observers seems at present to be in favor of the two following propositions: (1) *Convulsion is a symptom*: (2) *Convulsion is the result of a deficient supply of healthy blood in the brain and spinal cord.*

The object of the remainder of this paper is to elucidate these two propositions.

Boerhaave, a hundred and fifty years ago, gave the loss of too much blood, as one of the causes of convulsions, and when such is the case, "the cure is performed," he states, "by filling the vessels again with a soft, friendly and liquid aliment given in small quantity, but often, and "by stopping at the same time the loss of blood."

Helvetius, a contemporary and opponent of Boerhaave, says: "The necessity of a due proportion between the fluids and solids, evidently shows, that 'tis to the want of this, the convulsions and other symptoms in hæmorrhagies are to be ascribed. The plainest instance that can be given of this, is that of a dog or any other animal from whom an excessive quantity of blood has been taken."

Cullen maintains that "there are certain powers of collapse, which in effect, prove stimulants, and produce epilepsy," mentioning hæmorrhage as the first of these causes which he termed "indirect stimulants." Says Cullen in this connection; "that the same hæmorrhagy that produces syncope, often at the same time produces epilepsy is well known, and * * * * hæmorrhagies occurring to such a degree as to prove mortal, seldom do so without first producing epilepsy." (Under the general term "epilepsy," Cullen included, not only the disease known to us as epilepsy, but also certain other convulsive disorders, such as convulsions of children, hysterical and puerperal convulsions).

John Brown *the* stimulator of the last century, who rebelled against the bleedings, pukes and purges of Cullen, his followers and contemporaries, bringing down the maledictions of the whole army of depletors on his head, said: "Tremor, convulsion and every affection comprehended under it, are to be imputed to debility." Again in speaking of spasmodic and convulsive diseases, "Instead of arising from an over proportion of the blood and excessive vigor," says Brown, they depend "upon an under proportion of that fluid, and other causes of debility, and are to be cured, not by bleeding and other evacuations, but by filling the vessels and restoring the strength of the whole system."

Although Brown was considered by the then "Regulars," an "unfortunate genius," wild and visionary, as in fact he was in many things, not having the light of modern discoveries in physiology to guide him, it is interesting to consider how the medical profession have within the last hundred years constantly approached nearer and nearer his management of disease, if not adopting his particular treatment, at least agreeing with his opposition to the notions prevalent in his day, till now we are far ahead of him in substituting the *general support* which he so vehemently urged, for the murderous lancet and deadly potions in the way of "vomits and purges" with which his enemies hurried their patients out of the world.

No convulsion or spasm follows *congestion* of any part of brain or cord. The results of congestion are stupor, coma, paralysis.

In apoplexy the coma always preceeds the convulsions. First the brain is engorged with blood, and the man falls—this is the "shock," immediately the vessel or vessels burst, draining the brain of blood, producing *cerebral anœmia*, and the convulsion occurs at once. This theory that convulsion is dependent upon anæmia of the brain, has been made famous by Niemeyer.

Go to the shambles, observe the manner of dying—a hog is "stuck," as the blood drains away his strength fails, he staggers and falls, no convulsions yet, finally the great nerve centers are so drained of the life-giving fluid that they are unable to communicate a steady flow of nerve force to the muscular system—nature makes her last great effort, and throws the animal into convulsions which continue to the end. Sir Charles Bell and Marshall Hall, both maintained that the convulsions which occur when an animal is bled to death, are the result of loss of blood from spinal cord.

When an epilectic falls down he is deadly pale, not flushed as has been incorrectly stated. Pupils are dilated, a prominent symptom of cerebral anæmia. The tonic spasms occur first, throwing the blood into the cord to which they point. The clonic spasms, which point to the brain, follows in a few seconds. Then in another minute the fit is over.

In an epilictic the fault of his system, his disease, is a constant *tendency*, *liability*, to sudden excessive cerebro-spinal anæmia. From some cause or causes occasionally or periodically acting upon his system, the brain and spinal cord are suddenly deprived of their customary supply of blood. Convulsions and fibrillary quiverings follow as a result of this deprivation, the convulsions being nature's sudden spring to relieve the sudden dangerous cerebro-spinal anæmia. *The convulsion is not the fit proper—the anæmia is the fit, the convulsion the cure.*

To be sure the medulla has been found conjested and indurated in some old cases of epilepsy, where patients have died of intercurrent diseases. But this permanent congested condition is the result of repeated and long continued paroxysms, the vessels having become too greatly weakened to resist the force of the circulation during the fits. This chronic pathological condition is the *result* of paroxysms, not the *cause* of them.

There may be congestion and anæmia at the same time—the engorgement may be so great as to disable the capillaries from performing any function at all, paralyze them, or the congestion may continue so long, even if the capillary function is not arrested, that the blood is exhausted of all its vital principles, and charged with the natural waste instead. In either case there is virtually anæmia, for exhausted blood in healthy tissues, or good blood in paralyzed tissues, amounts to the same thing as no blood at all in those tissues. Passive congestion, then confirms, rather than contradicts the influence that epileptic attacks have their origin in failure of circulation, not in increased action.

One of the triumphs of physiology is the discovery, that irritation of a sympathetic nerve produces capillary contraction with diminished blood supply in parts to which the irritated nerve is distributed. I suggest that "the irritation of worms and indigestible substances in the alimentary canal," which causes the well known convulsions in children, is, in reality, irritation of a portion of the sympathetic system of nerves, this irritation being reflected to the spinal cord through which the sympathetic is abundantly distributed, producing contraction of the capillary blood vessels in this nervous center, temporary anæmia necessarily following this physiological action. Nature at once springs the convulsion on the organism for the purpose of restoring the blood to the cord, in many cases overdoing the matter by rupture of a blood vessel or by otherwise injurying or destroying the structure or function of tissues necessary to life. So also may "irritation of the gravid uterus" be in fact irritation of sympathetic nerve filaments—the last element in the process being that most appaling sight, puerperal convulsions.

And epilepsy too, may not the primary fault be irritation, derangement, diseased action, call it what we may, of the sympathetic nerve, this irritation being at times so great as to produce such excessive cerebro-spinal anæmia as to endanger life, nature immediately springing to the rescue with convulsions, without which the patient would die?

Dr. Echeverria is the first so far as I know, to publish the statement that bromide of potassium produces cerebral congestion. I am specially thankful for this suggestion, as I have never been able to harmonize the *theory* of its being a cerebro-spinal sedative, with the *fact* of its remarkably favorable effects in epilepsy. This statement of so distinguished an authority on epilepsy, gives a clue to its "mode of operation." This theory accords well with his advice to combine coffee and strychnine with it in the treatment of epilepsy. I would further suggest that bromide of potassium, by allaying the irritation of the sympathetic nerves, diminishes their power over the capillaries, allowing the circulation of more blood in the spinal cord, the very condition that is desired in epilepsy and all other convulsions. And this is the way bromide of potassium acts as a "nervous sedative."

In his recent lecture on cerebral exhaustion before the R. C. P., Dr. C. B. Radcliff includes epileptiform seizures among the symptoms of that disease; and insists upon it, that these seizures, whether fully developed epileptic or epileptiform, are connected with a bloodless, rather a congested state of the brain; and fully corroborates what Delasiauve first noticed, and Trousseau insisted upon, that a corpse-like paleness of the countenance is the immediate precursur of the perfect form of epilepsy. Says Radcliff: "I can testify to the existence or a corresponding palor at the same time deep down in the eye, for on several occasions, while happening to be examining the eye with the ophthalmascope when an attack of *petit mal* has come on, I have seen the pupil dilate, and the vascular blush in the fundus become quite pale." Again, in speaking of spinal exhaustion, he states that it is difficult to diagnosticate the two diseases, cerebral exhaustion and spinal exhaustion, that they have many symptoms in common, and that "very many of the peculiar symptoms belonging to spinal irritation, will be found belonging to spinal exhaustion."

Dr. Seguin in his recent admirable lecture on the therapeutics of the nervous system, gives "epileptic loss of consciousness" as typical of anæmia of the brain, and recommends nitrite of amyl at the moment *the spasm of the vaso motor nerve commences.*

Dr. Crichton Brown, in experimenting with nitrite of amyl, found that this powerful and rapid stimulant will usually prevent epileptic seizures when inhaled immediately premonitions of the fit are felt.

Dr. Robert Barnes, in his recent Lumleian lectures on convulsive diseases of women, does not support the doctrine advanced in this paper, but teaches that in the pregnant state there is what he calls "exalted central nervous erethism," "exalted nervous irritability," "excessive centric irritability" &c., but often gives the causes of this "irritability," "erethism," "excitability," in such terms as this: "some alteration of the blood," lowered or impoverished condition of the blood," indeed he gives "lowered or impoverished condition of the blood" as one of "the two great factors in the production of these diseases." The other "great factor" he names "exalted nervous irritability under the stimulus of the reproductive function." He has only to go a step further, and that a very short one—to say that the great factor" "lowered or impoverished condition of the blood," is the cause of the other "great factor," "exalted nervous irritability," and he is on our side.

Dr. Moreland states, that acute anæmia marked by the occurrence of convulsions and coma, leaves the brain anæmic.

Dr. Loomis recently read a paper before the N. Y. A. M., on acute uremia, in which he strongly advocates the hypodermic use of morphia for the convulsion, reporting several cases immediately relieved by the morphine.

The most severe local spasms that I ever witnessed, occurred in 1871, in the right lower extremity of a young married lady suffering from spinal anæmia, with atrophy of the limb, which had existed (the atrophy) since a fall in childhood. These spasms were almost sure to occur with the catamenia, and were always immediately relieved by hypodermic of morphia. My theory is that the part of the cord supplying nerves to the atrophied limb, was specially anæmic, badly nourished. The menstrual flow occurring quite profusely in a feeble, anæmic woman, suddenly drained the cord—the point of local malnutrition specially feeling the loss. Nature attempted to restore the blood to the part by throwing the limb into convulsion. (The spasms were tonic). I assisted nature with the morphine to produce congestion in the part at fault. As soon as this was accomplished the spasms ceased. Now what is the first effect of opium on the system? Dr. Crump decided from experiments in 1793, that any one might determine for himself by counting his own pulse under a dose of opium, that in its first operation it is a stimulant, at least to the circulation. Dr. Wood, our own great therapeutist and pharmacologist says: "The operation of opium upon the nervous centers I maintain to be essentially stimulant." "When a part is stimulated it receives an additional supply of blood, and the supply increases with the stimulation. Now the first effect of the blood entering a part is to supply the means for an increase of its healthy function; a large supply interferes with the due action of the part, through the excitement may still continue, a still further quantity overwhelms it and impairs or suspends altogether the exercise of its functions."

The anæmia may be so excessive as that a proper supply of blood may not be sufficient to enable the part to renew its functions.

The convulsions may be so violent or frequent as to produce such sudden and excessive congestion as to "interfere with the due action of the part," "overwhelm it," "impair or suspend altogether the exercise of its functions," as Wood says. In this way convulsion may cripple or kill. Here is presented the indication, in some rare cases, for bleeding, to *prevent* undue congestion, to *prevent* overwhelming nerve centers, to *prevent* apoplexy—not to prevent or to cure the convulsion.

Dr. Loomis is right in using morphia to produce immediate congestion of brain and cord. Others are right in using chloral for the same purpose. Our old-time friends are occasionally right in bleeding their patients with convulsions—not to stop the convulsion, but to stop its "running riot" to destruction. But they bleed to *relieve* undue congestion, not to *prevent* it—bleed on the theory that the congestion is the cause of the convulsion, when, according to the theory presented in this paper, the convulsion is the cause of the congestion.

I have in mind Mrs. R., a large, fleshy woman of 270 pounds, whom I have attended in ante-partum convulsions near the close of two parturitions. First, in Dec., 1871, she had three convulsions previous to my arrival, and one. terribly severe and long continued, after I entered the house before anything was done. The treatment at this time was all suggested by an older medical friend who accompanied me. Chloroform was given at once, during administration of which the fit ceased. When it was over she was bled freely, and given chloral gr. xv. every 15 minutes till asleep. She had no more convulsions. The chloral was continued for several days, till after still-born child was delivered in the natural way. Our ancient friends may well say, that, in this case, the bleeding and chloroform are as like to have been the saving means as the chloral. But in the last attack, Sept. 1873, I arrived sooner. I found her on the floor, just recovering consciousness from her first convulsion. Tongue was bitten and she was otherwise bruised by the fall. I commenced at once to give her large doses of chloral before removing her from the floor, repeating the chloral every ten minutes till she was asleep. This was the only treatment. *She had no more convulsions.* After three days, the fœtus, being dead, was delivered artificially.

Prof. Lusk says in a recent clinical lecture on puerperal convulsions, referring to bleeding: "Its virtue lies, it is to be remembered, purely in the fact that in the face of an immediate threatening danger, a little time may be gained in its employment." He states in this lecture that Traube and Rosenstein believe puerperal convulsions to be due to anæmia of the brain, although for himself he thinks that "probably we are nearest the truth, when we adopt the view that causes of puerperal convulsions are multiform."

